# An Analysis of Notch Toughness of Electron Beam Powder Bed Fused (EB-PBF) Ti-6Al-4V in Relation to Build Orientation and Mechanical Properties

**DOI:** 10.3390/ma19030524

**Published:** 2026-01-28

**Authors:** Mohammad Sayem Bin Abdullah, Vidit Tambi, Aditya Koneru, Dwayne Arola, Mamidala Ramulu

**Affiliations:** 1 Department of Mechanical Engineering, University of Washington, Seattle, WA 98195, USA; sayemab@uw.edu (M.S.B.A.);; 2 Department of Materials Science and Engineering, University of Washington, Seattle, WA 98195, USA

**Keywords:** additive manufacturing, anisotropy, Electron Beam Powder Bed Fusion (EB-PBF), notch toughness, prior beta grains, Ti-6Al-4V

## Abstract

A comprehensive analysis of the notch toughness of Electron Beam Powder Bed Fused (EB-PBF) Ti-6Al-4V was conducted, which focused on the influence of build orientation and correlations with key mechanical properties. Horizontal and vertical specimens were fabricated with optimized process parameters and reused powder. The microhardness and microstructure of the metal were examined and both profilometry and scanning electron microscopy were used in evaluating the fracture surfaces. Results showed that the metal with vertical build orientation absorbed ~46% higher impact energy than the horizontal orientation due to crack propagation perpendicular to the prior-β grains, lower microhardness, and greater ductility. The importance of ductility to the vertical specimens was evidenced by greater shear lip width (~51%) and height (~35%), greater shear lip length (~18%), and higher roughness of the fracture surface (~15%). Shear width measurements showed the highest correlation with absorbed impact energy. Overall, results show that the notch toughness of EB-PBF Ti-6Al-4V is dependent on the build orientation due to differences in microstructure and the bulk mechanical properties. The notch toughness is well correlated with tensile properties as well. Lastly, a framework for relating the notch toughness in dynamic loading and quasi-static fracture toughness for EB-PBF Ti-6Al-4V is proposed.

## 1. Introduction

Electron Beam Powder Bed Fusion (EB-PBF) is an additive manufacturing (AM) process that is used for the fabrication of components from a variety of metals. The EB-PBF process is used in the aerospace, automotive, and biomedical industries, among others, for rapid prototyping as well as for production of complex metal parts, including those of titanium [1,2]. There are some advantages of EB-PBF. Due to the high temperature of the build chamber, which generally exceeds 650 °C, and vacuum conditions, EB-PBF parts exhibit negligible residual stresses and no interference from gas flow, unlike in laser powder bed fusion (L-PBF) [3,4]. However, the roughness of EB-PBF parts is often greater due to larger powder diameter and beam diameter compared to those in L-PBF [5]. Machining is recommended for both processes while additional post-processing (i.e., heat treatment) is needed in L-PBF to remove residual stresses [6].

One of the major differences between PBF processes and traditional manufacturing processes (e.g., casting) is the greater number of process variables and uncertainties involved in PBF. Since PBF processes are performed layer by layer, the mechanical properties of fabricated metallic parts exhibit anisotropy due to variation in localized microstructure, process induced defects such as porosity and lack of fusion (LOF), and surface roughness. From a fracture mechanics perspective, the anisotropic impact behavior emerges from the coupled effects of crack-path direction, underlying microstructure, and the anisotropy of defect population and their alignment relative to the notch/crack plane. Indeed, the mechanical properties of EB-PBF Ti-6Al-4V varies with orientation and build location within the build space [7]. Specifically, the tensile strength, fatigue properties, and fracture toughness vary by as much as 15% [1,2,8]. While these properties are of substantial importance, they do not address the dynamic fracture resistance, where localized strains and strain rates are extremely high. The notch toughness under impact loading is an important mechanical property, which refers to the amount of energy that a material can absorb before rupture under dynamic loading. Charpy impact testing provides a convenient manner to explore dynamic toughness and notch sensitivity. These properties can be sensitive to microstructure and defects, which are concerns in PBF processes in general [9,10].

Ti-6Al-4V is widely used for stress-critical applications due to its high specific strength, superior fatigue, and fracture properties. It exhibits a strong balance between tensile strength and ductility due to Hexagonal Close-Pack (HCP) and Body-Centered Cubic (BCC) crystallographic structure [11]. While several studies have been reported on the notch toughness behavior of L-PBF Ti-6Al-4V [12,13,14,15,16,17,18,19,20], few have been reported on EB-PBF Ti-6Al-4V. Hrabe et al. studied the effects of internal porosity and crystallographic texture on the notch toughness of EB-PBF specimens in the as-built condition and after Hot Isostatic Pressing (HIP) considering horizontal and vertical orientations [21]. Results showed that the HIP treatment reduces internal porosity and increases the α lath thickness, which improved the absorbed energy. Their results emphasized that crystallographic texture and grain morphology influence energy absorption and that crack pathways crossing prior-β grain boundaries absorb higher energy. In another study performed on Ti-6Al-4V produced by EB-PBF, HIP improved the impact toughness and diminished effects of configuration on notch sensitivity [22]. Bruno et al. investigated the effect of build orientation (0°, 30°, 60°, 90°) on the microstructure and mechanical performance of Ti-6Al-4V parts fabricated by EB-PBF, and reported that horizontal builds exhibited lower ductility and toughness compared to vertical builds in Izod impact testing [23].

Aziziderouei et al. studied the effects of build orientation (considering 0°, 45°, and 90°) and lack of fusion (LOF) on the impact energy of Ti-6Al-4V produced by EB-PBF. They reported that the impact energy increased with effective angle between the notch direction and build orientation. Interestingly, LOF facilitated crack propagation when the notch direction was aligned with the build direction [24]. They also discussed the influence of microstructure on the crack paths and reported higher impact energy for vertical specimens. Jeffs et al. examined the effects of process parameters and build orientation on the notch toughness of EB-PBF Ti-6Al-4V fabricated from virgin powder [25]. Their results also showed that vertical builds exhibited higher impact energy due to epitaxial grain growth. The beam velocity and normalized energy density contributed to the impact energy due to their contribution to the printed microstructure. Grell et al. studied the effects of oxidation with powder reuse between 0.11% and 0.525% oxygen on the notch toughness of horizontal (XZ and XY orientations) and vertical (ZX) specimens after HIP treatment. They reported that notch toughness was correlated with tensile toughness and ductility. Their results showed that the differences in energy absorbed with build orientation were most prominent in the lower oxidized condition (below 0.14%); vertical specimens absorbed the highest amount of energy [26]. At higher oxygen content levels, the difference still exists; however, it is reduced due to increased oxygen content since interstitial oxygen can strengthen α-Ti through resistance to the motion of screw dislocations [26]. The effects of orientation and HIP disappeared when oxygen content increased beyond 0.5%.

Kazachenok et al. compared the impact toughness of EB-PBF, L-PBF, and electron beam freeform fabricated Ti-6Al-4V with wrought material. As expected, they reported lower impact energy absorption of the PBF materials than wrought material [27]. Soundarapandiyan et al. conducted two studies on the impact energy absorption of EB-PBF Ti-6Al-4V [28,29] via Charpy impact. In the first study performed on as-built EB-PBF Ti-6Al-4V with a machined notch, an increase in energy absorption was noted after HIP and Solution Treatment and Aging [29]. In a following study, the impact energy absorption of EB-PBF Ti-6Al-4V specimens showed minimal decrease with rising oxygen content since the content varied within from 0.08% to 0.10% [28].

Specimen geometry is an important factor to consider. The specimen geometries in References [24,25,26,27] are consistent except for the horizontal specimen in Reference [25]. The specimens in References [21,21,22] are smaller (24.13 × 4.83 × 4.83 mm with a 0.7–0.9 mm deep V-notch), and in References [28,29] are larger (120 × 30 × 30 mm with a similar 2 mm deep V-notch at the center). However, the fundamental findings, including the trend in energy absorption and crack propagation depending on orientation, remain the same. Summarizing the existing body of work, the microstructure of EB-PBF Ti-6Al-4V, often characterized by α + β phases and porosity, can cause structural anisotropy due to layer-wise processing, which can significantly influence the impact toughness of Ti-6Al-4V. Furthermore, HIP can improve the notch toughness of the alloy but is highly dependent on the treatment temperature and pressure.

Most of the previous research on the notch toughness of EB-PBF Ti-6Al-4V has interpreted trends in the impact energy to failure without mechanical property data or supporting fractographic evaluation. Optical profilometry is an effective method for analyzing the fracture surface morphology, both qualitatively and quantitatively, to achieve a mechanistic understanding. Hence, a detailed evaluation of fracture surface morphology of EB-PBF Ti-6Al-4V specimens after Charpy impact testing is necessary, including an identification of the key features. While the existing literature contains scanning electron microscopy-based qualitative analysis of fractured surfaces, and discusses the role of pores and lack of fusion with respect to the impact of EB-PBF Ti-6Al-4V [21,22,23,24,25,26,28,29], only Jeffs et al. [25] presented quantitative fractographic measurements, namely shear lip width. Notch toughness is reportedly a function of ductility and the dependence is often reflected through characteristic features of the fracture surfaces [30]. Within this mechanics framework, the present work is positioned as a study of dynamic fracture anisotropy in EB-PBF Ti-6Al-4V, and provides extensive quantitative details of the fractured surface resulting from impact toughness experiments, where differences in impact energy behavior are linked to build direction, fracture surface topography, microstructural and microhardness measurements, and reused powder-condition considerations, providing fundamental insight into how process and feedstock-dependent parameters interact with anisotropic microstructures to control energy dissipation under high strain rate impact loadings. Furthermore, discussion in the literature concerning the relationship between impact toughness and mechanical properties is limited to hardness [23,26,28]. To strengthen the interpretation, the effects of build orientation were evaluated in relation to surface topography, indentation hardness, tensile properties, and microstructure, including predominate process-induced defects. Based on these findings, their contribution to the notch toughness of EB-PBF Ti-6Al-4V is discussed.

## 2. Materials and Methods

### 2.1. Specimen Fabrication

**Figure 1:**
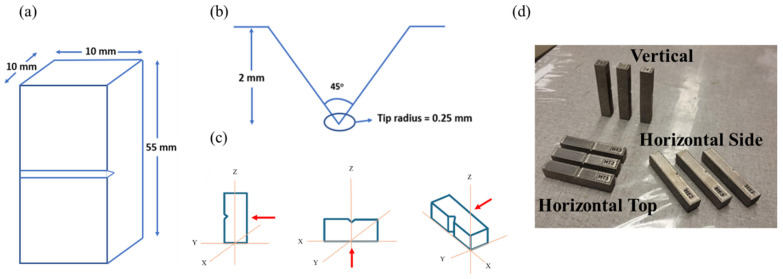
Details of the specimens. (**a**) Specimen dimensions (not to scale); (**b**) notch dimensions; (**c**) notch orientations with respect to the build plate orientation system (red arrow indicates impact direction); (**d**) example of selected fabricated specimens for each orientation.

Charpy impact specimens were built in accordance with ASTM E23 with three different orientations, including horizontal side (HS), horizontal top (HT), and vertical (V), as shown in Figure 1c [31]. Ten specimens were built, with each orientation in a single build, all with standard geometry (10 × 10 × 55 mm), notch depth of 2 mm, interior angle of 45°, and a notch-tip curvature of 0.25 mm (Figure 1). The position and orientation of the specimens inside the chamber were virtually modeled with 6 mm support structures in Materialize Magics software (Version 21.11). The build was completed on an ARCAM A2X EBM machine (Molndal, Sweden) operated with Version 5.0.64 control software, and using the default parameters for Ti-6Al-4V. The default build parameters included a beam current of 15 mA, max current of 20 mA, beam scan offset of 25 mA, beam speed of 4530 mm/s, speed function of 45, and preheat temperature of 650 °C, after [32,33]. Gas-atomized spherical Ti-6Al-4V powder with 21 prior build cycles has been utilized in this build. The nominal average particle diameter was approximately 50 µm, with most of the powder (>90% of particles) in the range of 45–120 µm. In the EB-PBF process, unused powder from the powder cake is recollected by the powder recovery system (PRS) and sieved after the completion of each build. Additional powder was only added to the existing powder volume when it was not enough to complete the next build. The initial mass of virgin powder was 100 kg. After the 12th build cycle, additional virgin powder was added at the following cycles: 50 kg at the 13th build, 10 kg at the 16th build, 1.5 kg at the 19th build, and 10 kg at the 22nd build (the present build). The oxygen content of the 19th build, at 0.242%, was measured at Fort Wayne Metals (Fort Wayne, IN, USA) according to ASTM E1409 [34]. The oxygen content shows consistency with historic data compiled by Derimow and Hrabe for this build cycle and powder replenishment strategy [35]. The powder chemistry was not tracked after every build cycle for this powder. However, in our previous work, variation in oxygen content was extensively tracked after every build cycle with no addition of virgin powder between each build. The 20th cycle for that research had an oxygen content of 0.269%. Thus, the oxygen content in the powder used to build the impact specimens is approximately equal to that of the 20th cycle from our previous work. The weight percentage composition of that feedstock is presented in Table 1 [36].

Using a similar approach, Borelli et al. were able to control severe oxidation in the EB-PBF process for Ti-6Al-4V [37]. The powder had experienced repeated thermal cycling during EB-PBF processing, mechanical handling through powder recovery system (PRS) operations, and intermittent atmospheric exposure during chamber opening, powder transfer, and sieving. The specimens were tested in the as-built condition without any additional post-processing. Tensile properties of the metal fabricated with this powder using the same ARCAM A2X machine and build parameters are listed in Table 2 [30,36,38].

**Table 1:** Weight percentage composition of the 20th build cycle for Ti-6Al-4V [30,36,38].

Build	Al (wt%)	V (wt%)	O (wt%)	N (wt%)	Fe (wt%)	H (wt%)
20	5.54	3.40	0.269	0.027	0.26	0.0012

**Table 2:** Tensile properties of EB-PBF Ti-6Al-4V specimens after 20 cycles [30].

Orientation	Elastic Modulus (GPa)	Yield Strength (MPa)	Ultimate Tensile Strength (MPa)	% Elongation
Horizontal	102 ± 0.8	927 ± 18.9	1010 ± 21.8	4.3 ± 0.25
Vertical	113 ± 5.9	939 ± 7.5	1070 ± 11.3	9.18 ± 0.99

### 2.2. Charpy Impact Experiment

**Figure 2:**
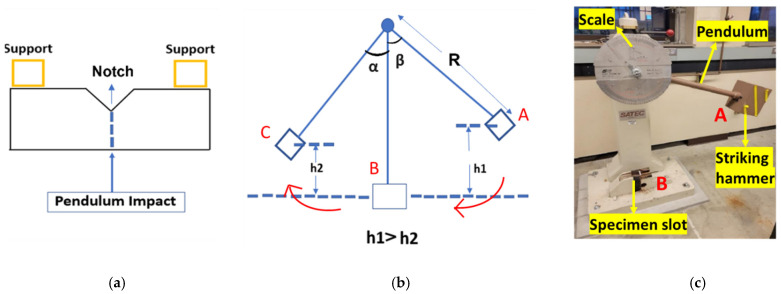
Pictorial representation of Charpy impact testing: (**a**) schematic of the top view at the specimen holder; (**b**) schematic of the test set up; (**c**) actual test set up and tester with a specimen loaded. A, B, and C, respectively, represent initial height, specimen position, and the maximum height the pendulum reaches after the impact. Arrows show the direction of pendulum movement.

The Charpy impact test quantifies the notch toughness of metals under impact loading. The experiments in this investigation were performed using a Satec SI-1K3 (Warren, MI, USA) adhering to the ASTM E23 standard [31]. Figure 2 outlines the experimental set up for the Charpy impact test. The instrument was calibrated prior to each experiment, as required for accurate measurements. The Charpy impact test evaluates the energy absorbed during fracture, which is calculated according to the following equation:

(1) $$E\;(J)\;=\;E_{i}\;-\;E_{f}$$

where E_i_ and E_f_ are the initial potential energy of the pendulum and the final energy after impact loading, which are given by the following equation:

(2) $$E_{i}\;=\;mgR\;\left(1\;-\;cos\;\beta \right),\;and\;E_{f}\;=\;mgR\;\left(1\;-\;cos\;\alpha \right)$$

In Equation (2), m is the hammer mass, g is gravitational acceleration, and R is the pendulum radius of swing [20].

### 2.3. Optical Profilometry

An optical profilometer (Keyence, Model VR-3100, Osaka, Japan) was used to characterize the surface topography of the EB-PBF Ti-6Al-4V specimens after impact testing. Optical images and 3D topography of the fracture surfaces were captured at between 12× and 180× magnification. Standard surface roughness parameters were calculated for each specimen, including average area roughness (Sa), maximum height variation (Sz), and root mean square height (Sq). In addition, average peak height (Sp) and average valley depth (Sv) parameters were estimated [39]. Topography maps of the fractured specimens were created to support identification of the dominant characteristics in the fracture surfaces. The entire fracture surface excluding the shear area was considered as the reference plane in profiling the surface topography.

### 2.4. Shear Area Analysis

The shear area for each fracture surface was measured from high-resolution color maps obtained using the Keyence optical profilometer (Model VR-3100, Osaka, Japan) and post-processing using ImageJ software (Version 1.46r, Bethesda, Rockville, MD, USA). For each fracture surface evaluated, the total shear area was estimated from the sum of the left-side and right-side shear regions. To account for potential variations in measurements and to minimize errors, average shear area was calculated considering both sides of each broken specimen. Shear areas were then averaged across the 10 specimens of each build orientation to reflect the effects of build orientation on the fracture behavior.

**Figure 3:**
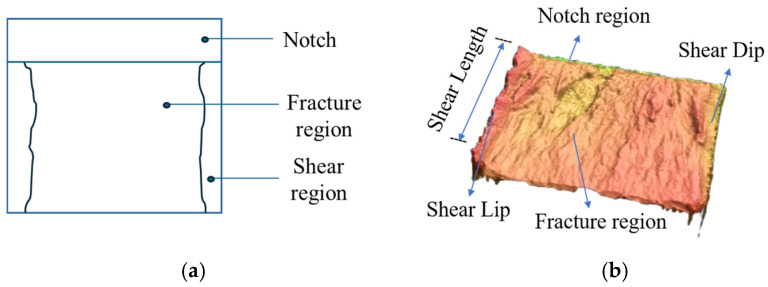
Details concerning quantitative analysis of fracture surface morphology. (**a**) Cracking and shearing regions of a fracture surface; (**b**) surface morphology of the fracture region.

The measured shear area is the projected area as seen from the top view of the fractured surface rather than the actual 3D shear area. Using the projected shear area, shear angle, and trigonometric principles, the actual shear areas and, consequently, the shear widths of the specimens were calculated, which is a more comprehensive reflection of the shear mechanisms. In addition, shear lip dimensions, including length and height, were calculated from the 3D topography, as shown in Figure 3. Jeffs et al. also used shear lip width to discuss the fracture behavior of EB-PBF Ti-6Al-4V [25]. Two more indices have been introduced and measured in this study, namely Maximum Continuous Shear Lip Length (MCSL) and Maximum Continuous Shear Dip Length (MCSD). MCSL and MCSD are defined as the maximum length of the continuous shear lip and shear dip in a fractured surface. The shear dips are the recessed regions on the opposite side of the fracture surface relative to the shear lips. In addition, the shear angle of the shear lips was calculated considering the vertical plane as a reference [40].

### 2.5. Microstructure and Microhardness Analysis

Selected specimens were sectioned, mounted, and polished to evaluate the microstructure and measure the microhardness. Out of the 30 total specimens, 11 representative specimens were evaluated. One half of each fractured specimen was mounted in a graphitic mounting compound using a conventional mount press. Polishing was performed using an EcoMet 30 semi-automatic polisher (Buehler, Lake Bluff, IL, USA). The process began with sequential grinding using silicon carbide (SiC) papers of 240, 400, 600, 800, and 1200 grit. Each grit was applied for 3 min at 300 RPM under a polishing load of 20 N per mount, with continuous water coolant. Fine polishing was performed using a 1 mL DiaLube diamond suspension (~3 μm particle size) for 5–6 min without additional water. To achieve a mirror-like surface, a final chemical polishing step was performed using a mixture of 0.5 mL NH_4_OH and 2 drops of H_2_O_2_ in 10 mL colloidal silica (~0.05 μm silica particle size) for 5 min. The microstructure was revealed by etching the final polished specimens with Kroll’s reagent (2% HF, 6% HNO_3_, and 92% H_2_O) for 8–12 s. Optical images were then captured using an optical microscope (Nikon BXZ5150, Tokyo, Japan) at magnifications of 50×, 100×, 200×, 500×, and 1000×.

To estimate microhardness, a micro-indenter (LM247AT, LECO Corp., St. Joseph, MI, USA) equipped with AMH43 software (Version 1.96, LECO Corp., St. Joseph, MI, USA) was used, following ASTM E92 standards [41]. Each specimen underwent at least 15 indentations, with an applied force of 100 g for 13 s per indentation. To comply with ASTM E92 guidelines, the indentation sites were examined before and after testing, ensuring a minimum spacing of 2.5 times the indentation diagonal (2.5 d) between adjacent impressions to prevent interference.

### 2.6. Scanning Electron Microscopy

**Figure 4:**
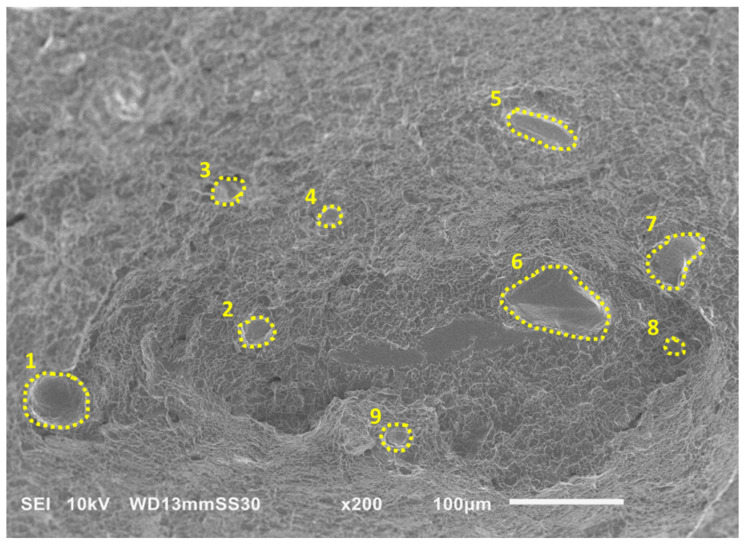
Calculation of pore area and porosity percentage using ImageJ software. The yellow dashed lines (1–9) outline the lack-of-fusion (LOF) voids and pores visible to the naked eye.

The detailed features on the fractured surfaces were evaluated by scanning electron microscopy (SEM) using a commercial instrument (JEOL JSM 6010PLUS/LA, JEOL Ltd., Akishima, Japan). Prior to performing SEM, the specimens were cleared of any debris with a nitrogen gas gun. Images were obtained at between 30–400× magnification. Two images of each of the three build orientations were analyzed using the ImageJ software (e.g., Figure 4) to estimate the percentage porosity with respect to the total fractured surface area.

## 3. Results

### 3.1. Impact Energy

**Figure 5:**
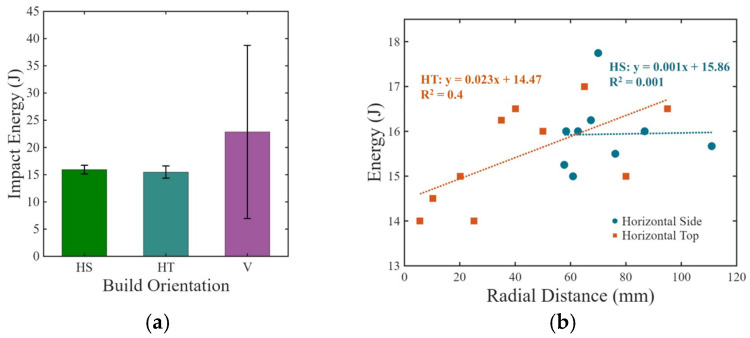
Impact energy variation (**a**) for the three different build orientations, and (**b**) as a function of radial distance from the center of the build plate for the horizontal orientations.

The impact energy to fracture of the EB-PBF specimens is presented in Figure 5a for all three build orientations. The average impact energy for the horizontal side (HS), horizontal top (HT), and vertical (V) orientations is 15.9 J ± 0.8, 15.5 J ± 1.1, and 22.9 J ± 15.9, respectively. As evident from the distribution, the vertical orientation exhibited over 45% higher average absorbed energy than the two horizontal orientations. Although the HS specimens exhibited slightly higher (~3%) average impact energy than the HT group, this difference is small relative to the experimental scatter and the standard deviations. A *t*-test comparing HS and HT indicates that the difference is not statistically significant (*p* = 0.31). Therefore, the two horizontal orientations are considered mechanically equivalent within experimental uncertainty.

The importance of build location on the impact energy of the horizontal specimens is presented in Figure 5b. Overall, there was limited change in energy to fracture with radial distance for the HT specimens and no trend observed in the HS specimens. When comparing notch toughness, the V specimens exhibited a higher average toughness of 0.285 ± 0.188 J/mm^2^, followed by the HS and HT top specimens, which had an average toughness of 0.199 ± 0.009 J/mm^2^ and 0.193 ± 0.013 J/mm^2^, respectively. Notch toughness follows a similar trend to impact energy absorption since the geometry for each group was similar.

### 3.2. Topography and Quantification of Shear Region

**Figure 6:**
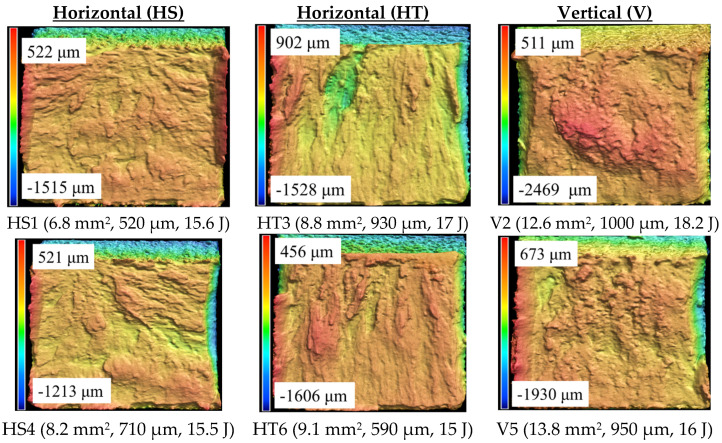
Surface topography of the fractured EB-PBF Ti-6Al-4V. The height scale is overlayed on the left edge on each color plot with maximum and minimum height values indicated. The measured shear lip area, average shear lip height, and absorbed impact energy are listed inside parentheses.

Figure 6 shows the surface topography of six representative fractured specimens, including the three build orientations. Shear lips and shear dips are clearly visible on the edges of the specimens. The measured shear lip area, average shear lip height, and absorbed impact energy are provided in the legend below each specimen. The shear areas were distributed on the fractured specimens either in the form of shear lips or shear dips. The shear lips were either continuous or discontinuous. All the fractured surfaces appear rugged, with valleys and peaks on the fractured surfaces. However, the height variation on the vertical specimens is comparatively much higher than that of the horizontal specimens.

**Figure 7:**
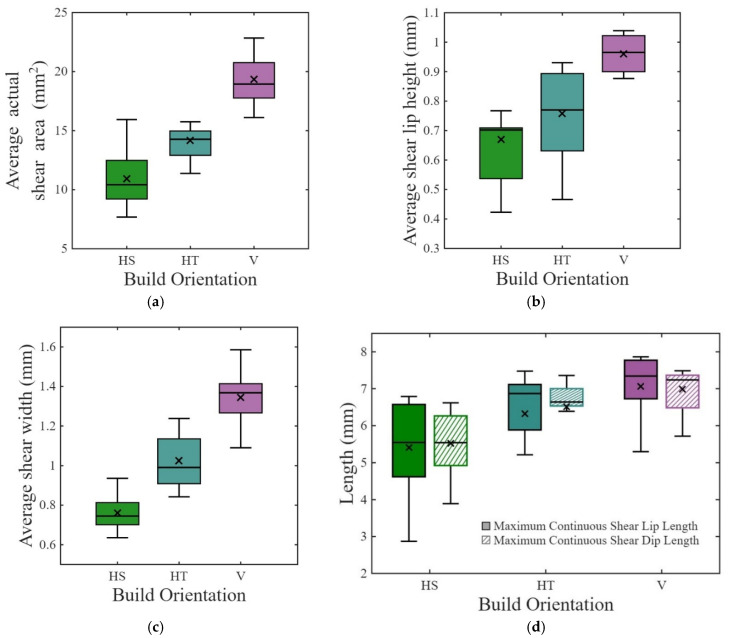
Distribution of average measured (**a**) shear area, (**b**) shear lip height, (**c**) shear width, and (**d**) MCSL and MCSD lengths for the three build orientations.

Quantitative results for the fracture surface shear area measurements are presented in Figure 7a. Overall, shear area was most predominant for the vertical specimens, as is evident in Figure 7a. The cross-sectional area available to resist fracture beyond the notch was ~80 mm^2^. Of this area, approximately ~14%, ~18%, and ~24% of the total fractured area was attributed to the shear region for the HS, HT, and V specimens, respectively.

Figure 7b presents the average shear lip height measurements with respect to build orientation. The areas affected by the dominant shear regions at the edges and the notch were excluded in these measures. As is evident, the shear lip height for the vertical specimens is significantly higher than the height for the two horizontal orientations. Overall, the average shear lip height of the vertical specimens is ~35% higher than that of the horizontal specimens.

The average shear lip width measurements for the three orientations are presented in Figure 7c. The average shear widths for the HS, HT, and V specimens are 0.76 mm ± 0.082, 1.02 mm ± 0.131, and 1.34 mm ± 0.125, respectively. The vertical specimens showed over 50% greater shear lip width (51%) compared to horizontal specimens combined of HS and HT. The shear angles were also calculated and found to be roughly 43.5°, 42.0°, and 44.6° for the HS, HT, and vertical specimens; the overall average was 43.4° considering all three orientations.

The average Maximum Continuous Shear Lip Lengths (MCSL) for the HS, HT, and V specimens are shown in Figure 7d and were estimated to be 5.4 mm, 6.3 mm, and 7.1 mm, respectively. The continuous shear lip lengths account for approximately 54% (HS), 63% (HT), and 70% (V) of the specimen widths, respectively, for these three orientations. As expected, the average Maximum Continuous Shear Dip Lengths (MCSD) for the HS, HT, and V specimens were very consistent with the MCSL measurements, with values of 5.5 mm, 6.5 mm, and 7.0 mm, respectively (Figure 7d).

Table 3 presents the average surface roughness parameters obtained for the flat fracture surfaces. These values represent the roughness posed by the surface asperities, excluding the shear area and notch area. The mean Sa values for the HS, HT, and V specimens are 18.1 μm, 19.1 μm, and 21.4 μm, respectively. Similarly, the mean Sv values for the HS, HT, and V specimens are 124.1 μm, 175.5 μm, and 164.2 μm, respectively. Overall, the roughness of the fracture surface of the vertical specimens is higher than that of the horizontal specimens. This agrees with the morphology evident in Figure 6 as the surface topography of the vertical built EB-PBF Ti-6Al-4V specimens appears more rugged than that of the horizontal specimens, suggesting higher ductility. Overall, the fracture surfaces of the horizontal specimens appear less rugged and comparatively flatter than those of the vertical specimens.

Table 4 represents the correlation coefficients of impact energy absorption with shear width, shear height, and MCSL, combining all orientations. Their respective correlation coefficients are 0.43, 0.04, and 0.31, respectively. Thus, among the shear features, shear width shows the highest correlation with energy absorption, followed by shear lip height. According to Table 4, the correlation coefficients of the impact energy absorption with Sa and Sq are 0.36 and 0.30, respectively, exhibiting moderate correlation.

### 3.3. Microhardness and Microstructure

Microhardness is a measure of the resistance to permanent deformation and for metals is often proportional to the ultimate tensile strength [42]. The average microhardness of the HS and HT specimens was determined to be 355.5 ± 2.9 and 362.3 ± 8.3, respectively. The average microhardness of the vertical specimens is 351.3 ± 3.8.

**Figure 8:**
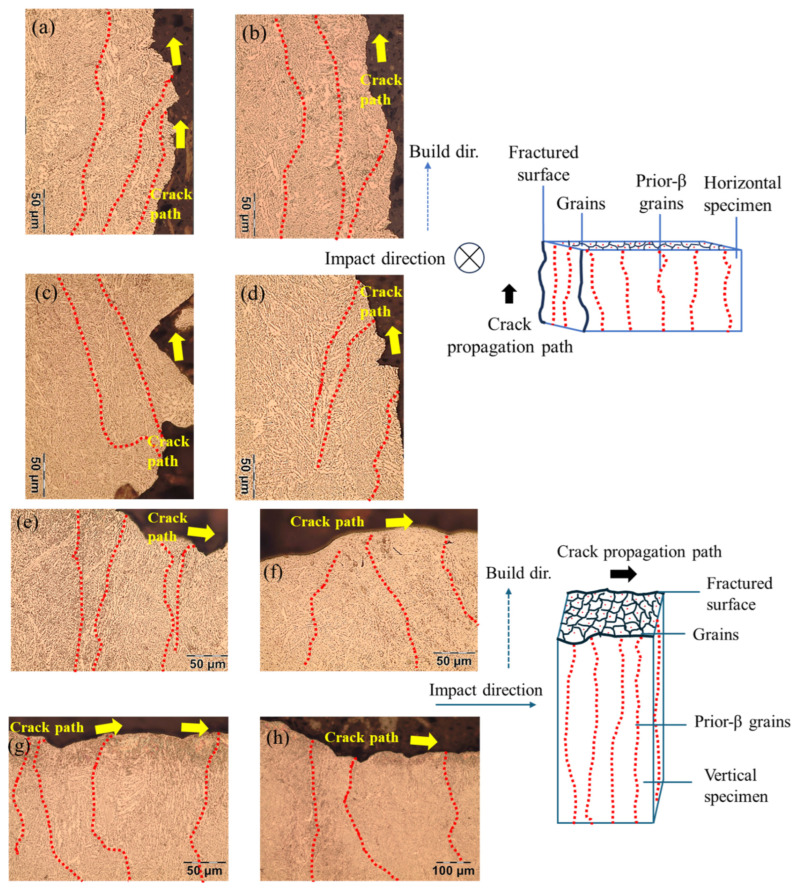
Sectioned views of the fracture surface, crack path, and prior-β columnar grains within the microstructure of EB-PBF Ti-6Al-4V (**a**–**d**) horizontal specimens and (**e**–**h**) vertical specimens. The prior-β grains are highlighted with red dashed lines. Schematics of prior-β columnar grains, crack direction, and fractured surfaces are provided for both orientations on the right of the microstructure images.

Figure 8 shows optical micrographs for sectioned surfaces of the horizontal and vertical specimens with annotation of the crack paths and columnar β grains. As is evident for the horizontal specimens, the crack travels predominantly along the prior β boundaries and not across. These findings align with previous research on Ti-6Al-4V fracture behavior [24]. In contrast, for the vertical specimens, the crack propagates through α and β colonies, crossing prior β grains orthogonal to the boundaries in the process, indicating trans-granular propagation.

### 3.4. Scanning Electron Microscopy

**Figure 9:**
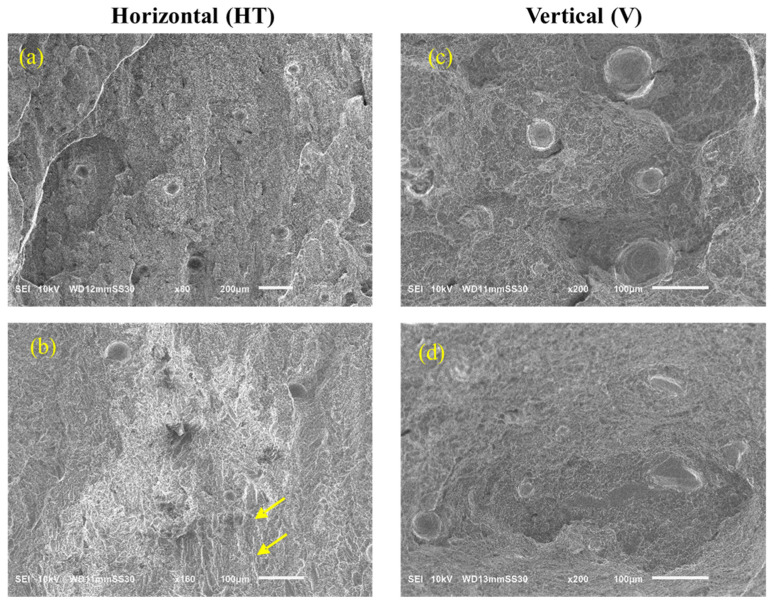
SEM images of fractured EB-PBF specimens with (**a**) horizontal (HT)—60×, (**b**) horizontal (HT)—160×, (**c**) vertical (V)—200×, and (**d**) vertical (V)—200× orientations. The yellow arrows in these images highlight the fracture flutes.

Results of SEM analysis of the fractured EB-PBF Ti-6Al-4V specimens with horizontal (HT) and vertical (V) orientations are shown in Figure 9 at multiple magnifications. The fractured surfaces exhibit process defects, including porosity and LOF voids. Porosity is evident for both the horizontal and vertical specimens as circular voids. The average porosity measurements from fracture surfaces of the horizontal and vertical orientations were 1.8% and 4.5%, respectively, indicating that the defect population in the vertical specimens was about ~2.5 times greater than the horizontal specimens. It is important to note that this does not represent the actual porosity within the entire volume, which would have been better analyzed using volumetric characterization techniques (e.g., µCT). The surfaces also exhibit micro-void coalescence such as fracture flutes [30], as highlighted with yellow arrows in Figure 9b. These flutes are elongated dimples and are common to HCP structures. They signify ductile fracture and develop from a reduction in slip in the HCP-α phase due to increasing oxygen content in the material [30]. These flutes tend to form on $\left\{10\underline{1}0\right\}<11\underline{2}0>$ along α–α and α–β grain boundaries and are also often formed in Widmanstatten microstructures due to the close packing of the α and β plates [43].

## 4. Discussion

The mechanical behavior of Ti-6Al-4V produced by EB-PBF varies with build orientation and was reflected in the bulk tensile properties, microhardness, and notch toughness. This section comprehensively discusses the influence of build orientation on impact energy absorption and notch toughness in relation to mechanical properties, microstructure and porosity, and fracture toughness along with limitations of the work.

### 4.1. Influence of Microstructure and Fracture Surface Topography

The notch toughness of the EB-PBF Ti-6Al-4V specimens was evaluated in an as-built condition and exhibited anisotropy with respect to build orientation. While there was minimal difference in the impact energy between the two horizontal orientations (HS and HT), the vertical specimens had over 45% higher average impact energy than the horizontal specimens (Figure 5). The crack path driven by dynamic fracture in the vertical plane involved interaction with the microstructure, i.e., the prior beta grain boundaries, that absorbed more energy.

**Figure 10:**
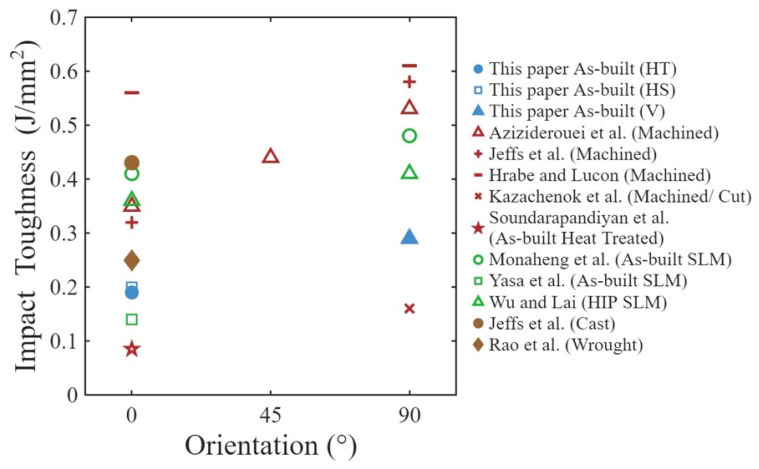
Broad comparison of notch toughness of as-built and machined EB-PBF Ti-6Al-4V with the literature [12,20,21,24,25,27,29,44,45].

According to the experimental results presented in Section 3.1, the impact toughness obtained in this study is comparable to that reported for the as-built condition in previous studies [23,25,26]. A comparison of the notch toughness values from this study with others reported for EB-PBF Ti-6Al-4V is shown in Figure 10. The overall average impact energy absorbed (18.1 J) resulting from EB-PBF is comparable to that for the Ti-6Al-4V alloy in wrought form (19.94 J) [44]. Considering the oxygen content, the energy absorption values for HS, HT, and V are close to non-HIP EB-PBF Ti-6Al-4V of similar geometry at ~0.275% oxygen content, with values of 10, 12, and 18 J reported by Grell et al. [26] and 13 J by Kazachenok et al. [27]. However, impact energy absorption is lower than expected from virgin powder (ranging between 20 and 50 J depending on orientation and notch direction) due to reduction in slip by increased oxygen content resisting dislocations [26,28]. As is evident in Figure 10, the impact toughness of EB-PBF Ti-6Al-4V is also comparable to LB-PBF and cast Ti-6Al-4V [20,45,46]. However, it is lower than values reported for EB-PBF Ti-6Al-4V in the machined and HIP condition, which warrants further discussion. The as-built condition of the metal had more surface asperities and larger apparent surface stress concentrations compared to the machined condition, thereby reducing its capacity for energy absorption. Clearly, the as-built surface condition leads to higher local stress concentration due to the external surface texture, which can result in lower energy absorption. Furthermore, the use of reused powder can cause further reduction in energy absorption as greater oxygen content tends to decrease the ductility in EB-PBF Ti-6Al-4V [30,38,47].

Results confirmed that build orientation is important to the impact energy absorption of the metal. The larger notch toughness of vertical specimens is due to the influence of microstructure, as reflected by the greater ductility and lower microhardness. The orientation of crack propagation in the vertical specimens is perpendicular to the columnar prior β grains. Crack deflection is promoted by their orientation, and the propagation ultimately occurs through more α-β grain boundaries. Moreover, the prior β-grains are bent and fractured transversely in the vertical specimens as driven by the orientation of impact loading (Figure 8). Crack deflection caused by α-β grain boundaries enhances energy dissipation by increasing the fracture surface area per unit crack extension; in the horizontal specimens, the crack propagates parallel to the prior-β grain, reducing the extent of crack deflection. Therefore, the impact toughness is greater for the vertical specimens. The difference in energy absorbed between the horizontal top face and horizontal side faced specimens is minimal due to similarities in crack propagation direction and prior-β grain directions. Regarding hardness, the average hardness of the vertical specimens was lower than that of the horizontal specimens. The rank of microhardness measurements between the horizontal and vertical orientations is consistent with the published literature [23,33,48]. For the vertical orientation, the specimen height is largest and the contact area with the build plate is smallest. Thus, the heat transfer rate is comparatively lower for the vertical specimens, resulting in lower cooling rates [23] and greater stored thermal energy [49]. A lower cooling rate results in decreased microhardness in the vertical specimens.

Results from optical profilometry showed that the vertical specimens had greater signs of inelastic deformation that accompanied fracture when compared to the horizontal specimens. The ruptured surface of the vertical specimens showed ~51% greater shear lip width and ~18% longer average continuous shear lips (combining for ~54% greater actual shear area); there was also ~35% greater shear lip height. Fracture surfaces of the vertical specimens exhibited ~15% higher roughness and more rugged topography. Shear width, actual shear area, and shear lip length are moderately correlated with energy absorption, where shear width showed a greater correlation than other features. While the correlation with shear width in our research is 0.59 for vertical specimens, Jeffs et al. [25] reported greater quantitative correlation for virgin powder fabricated specimens. The lower correlation is expected to result from the brittleness caused by oxidation [26]. Also, the fabrication parameters of Jeffs et al. [25] are different from this research, which can influence the variation as well. However, their study did not report shear lip height and length. Although shear lip height was ~35% greater for the vertical specimens, their correlation is not significant. This topic deserves further evaluation.

The vertical specimens exhibited higher average shear angles (44.6° ± 4.7) than the horizontal specimens (42.8° ± 7.2); the difference was not significant. The slightly lower shear angle of the horizontal specimens could result from more rapid crack propagation facilitated by extension along the prior-β grain boundaries. In a previous study on the fracture toughness of EB-PBF Ti-6Al-4V, Mojib et al. reported a smaller shear area for horizontal specimens than for vertical specimens [8]. Thus, the greater energy absorbed by the vertical specimens results from formation of wider and longer shear lip and a comparatively rugged fracture surface, which was facilitated by crack deflection at the crossing of prior-β grain boundaries. Overall, the energy absorption on impact is dissipated as plastic energy through ductile shear, with the remainder expended as surface energy associated with crack propagation [50].

### 4.2. Influence of Tensile Properties

**Figure 11:**
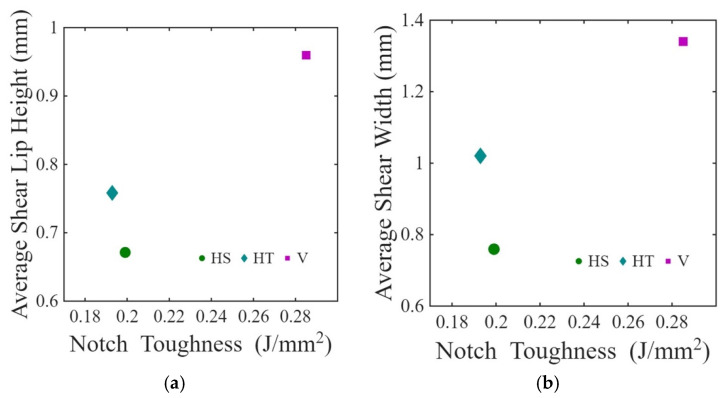
Distribution in notch toughness with respect to (**a**) average shear lip height and (**b**) average shear lip width.

According to previous related research [30], the percentage of elongation achieved by the vertical and horizontal EB-PBF Ti-6Al-4V specimens produced with the same powder is 9.18% and 4.30%, respectively (see Table 2), which agrees with the literature [23]. The vertical orientation has significantly higher tensile toughness (83 ± 17 MPa) than the horizontal orientation (51 ± 19 MPa) [38]. The vertical specimens have greater ductility and energy absorption due to the orientation-specific improvement in slip transmission between α + β colonies; the horizontal specimens exhibit less plasticity because of limited slip across prior β-grain boundaries [30]. Thus, both the tensile properties and hardness help interpret the orientation dependence in response to impact loading. Figure 11a,b shows the average shear lip height and shear width with respect to the notch toughness obtained from the experiments. Greater correlation of impact energy and shear width for vertical can be seen in Table 4. These results agree with those of Jeffs et al., who reported greater shear lip width in the vertically built EB-PBF specimens with respect to the horizontal orientation [25].

### 4.3. Influence of Porosity and Lack of Fusion

Porosity can influence the energy absorption and crack propagation of additive metals [21]. The Charpy impact test involves mode I loading, and the direction of crack propagation may align with the LOF voids [24,38]. Thus, notch toughness can be higher or lower than the average value depending on the interplay of LOF voids, porosity, and microstructural texture with crack propagation. The greater variation in notch toughness of the vertical specimens can be attributed to LOF void geometry, population, and loading mode [24,38]. Approximately 80% of the vertical specimens exhibited higher impact energy absorption (notch toughness) than the horizontal ones, which is expected to be driven by crack deflection and crack propagation perpendicular to the columnar prior-β grains. The remaining 20% of lower energy absorption may result from coupled interactions between porosity and variations in grain orientation [26]. However, that comment is admittedly speculative and requires further research.

The qualitative trend in fracture surface features between the vertical and horizontal specimens is consistent with our previous study in quasi-static loading of EB-PBF Ti-6Al-4V, which also involved reused powder with 0.189% oxygen content [8]. For 30 builds performed with Ti-6Al-4V fabricated with similar parameters in the same EB-PBF system, Schur et al. observed that porosity plays a limited role in ductility for the horizontal specimens, whereas vertical specimens show clearer dependence on porosity and lack-of-fusion voids. Similarly, Ghods et al. reported that vertical specimens underwent the largest decrease in ductility due to greater sensitivity to the larger lack-of-of fusion voids and associated stress concentrations. For similar reasons, the vertical specimens were expected to exhibit greater variation in impact energy to fracture. However, when compared to the mean value, the orientation-dependent microstructure plays a more dominant role than porosity in governing the overall trend in impact energy.

### 4.4. Proposed Relationship Between Notch Toughness and Fracture Toughness

In metals, the largest contribution to energy absorption is through plastic deformation. Both impact and fracture obey the minimum energy density criterion during crack initiation and propagation. Consequently, a quantitative relationship may exist between these two forms of toughness. Establishing such a correlation is useful since both the parameters reflect the competition between shear and flat fracture modes, as reported for high-strength steels by Li et al. [51]. Because both the Charpy and fracture toughness (*K*_IC_) specimens exhibit a transition from flat to shear fracture at the edges, Li et al. introduced shear lip width as a physical bridge between impact and fracture toughness. In that effort, the Charpy specimen’s shear width (*t_c_*) and the *K*_IC_ specimen’s shear width (*t_k_*) were related by the specimen-size correction, as follows:

(3) $$t_{k}=\;\delta t_{c}-\;\delta_{0}$$

where δ is the specimen-size factor and δ_0_ is the shear lip width related to crack initiation, which in this framework is treated conceptually rather than directly measured. This approach assumes that once shear lips are fully developed, their width remains constant along the fracture path.

According to the principles of fracture mechanics, plastic-zone size γₚ at the crack tip is proportional to ${\left(\frac{K_{IC}}{\sigma_{y}}\right)}^{2},\;$ where $\sigma_{y}\;$ is the yield strength, which can be expressed as follows:

(4) $$\gamma_{p}=\alpha {\left(\frac{K_{IC}}{\sigma_{y}}\right)}^{2}$$

Shear lips form in the near-surface region of plane stress where plastic deformation is largest. Their width provides a practical geometric measure that scales with the plastic-zone size at the crack tip. Since shear lip width represents the plastic-zone boundary, where $t_{k}\;\propto \;\gamma_{p}$ and β is a material constant, it can be written as follows:

(5) $$t_{k\;}=\;\beta {\left(\frac{K_{IC}}{\sigma_{y}}\right)}^{2}$$

For impact loading, Duan et al. [52] and Zhou et al. [53] established a relationship between Charpy shear lip width *t_c_* and intrinsic impact toughness α_c_ (≈αₖ for a 10 mm wide specimen), as follows:

(6) $$t_{c}=\;\gamma \left(\frac{\alpha_{k\;}}{\sigma_{y}}\right)$$

where γ is a constant and $\alpha_{k}$ is the intrinsic impact toughness of the material [52,53]. Variable $\alpha_{k}$ is a geometry-normalized impact toughness parameter that better reflects a material’s ability to absorb energy through plastic deformation during dynamic fracture. Substituting Equations (5) and (6) into Equation (3) eliminates the intermediate variable $t_{k}$ and $t_{c}$, linking fracture and impact toughness directly. The quasi-static plane strain fracture toughness and dynamic notch toughness are then correlated as follows:

(7) $${\left(\frac{K_{IC}}{\sigma_{y}}\right)}^{2}=\;\theta \left(\frac{\alpha_{k\;}}{\sigma_{y}}\right)-\;\omega$$

where θ is a specimen size coefficient and ω is the shear lip width parameter related to crack initiation, which are material constants.

In the presence of a crack, $\frac{K_{IC}}{\sigma_{y}}\;$ and $\frac{\alpha_{k\;}}{\sigma_{y}}\;$ represent the relative importance of crack propagation to plastic deformation in quasi-static loading and energy absorption to plastic deformation in dynamic fracture, respectively. The applicability of this framework to EB-PBF Ti-6Al-4V was assessed using fracture toughness data reported in prior studies with similar build orientations (14 total), EB-PBF processing parameters, and crack direction (Vidit [28] and Mojib et al. [8]). However, due to the differences in specimen geometry, powder reuse, and testing conditions, a one-to-one comparison is not possible. Furthermore, with limited data points in the present work, this framework is yet to be validated. However, it can provide a physically motivated basis to link dynamic energy absorption and quasi-static crack-growth resistance after accounting for yield strength and geometric effects, which would require validation and modification for future research on EB-PBF Ti-6Al-4V. Overall, the discussion is intended to be suggestive. Future work should attempt to reproduce the Li et al. [51] methodology under fully matched conditions (i.e., consistent specimen geometries, crack orientations, powder/reuse state, and post-processing) and include sufficient replicate testing and shear lip measurements to determine *θ* and *ω* for EB-PBF Ti-6Al-4V and to assess the robustness of the proposed scaling.

Although the findings of this investigation provide new understanding concerning the notch toughness of Ti-6Al-4V resulting from EB-PBF, there are some limitations that should be considered. Of key importance, this research explored metal behavior in an as-built condition only without further treatment. In addition to the value of including more build orientations and a larger number of specimens to increase statistical power, the investigation should be extended to consider machined condition and other post-processing treatments. Perhaps most important is inclusion of Hot Isostatic Pressing to remove the contribution of internal porosity. Furthermore, although the trend in porosity is consistent with extensive porosity measurements reported in [8,38], porosity measurements were limited in this study. Further work involving a detailed evaluation of the microstructure is warranted. Overall, results show that a design for EB-PBF focused on dynamic toughness should orient components such that the plane of maximum principal stress will be perpendicular to the build orientation and the elongated prior β grains.

## 5. Conclusions

An experimental investigation of the notch toughness of Electron Beam Powder Bed Fusion (EB-PBF) Ti-6Al-4V fabricated from reused powders was performed with respect to build orientation in as-built conditions. The importance of the bulk mechanical properties and microstructure were evaluated. According to the findings, the following conclusions are drawn:

The average notch toughness of the horizontal and vertical specimens was 0.20 J/mm^2^ and 0.29 J/mm^2^, respectively. The vertical specimens showed greater variation due to the synergic effects of larger ductility, porosity/lack of fusion, prior β grain boundary contributions to crack growth resistance, and lower microhardness.

The fracture surface topography of EB-PBF Ti-6Al-4V can be categorized into a shear region and fracture region. The shear region was characterized by shear area, shear lips (height, width, and angle), and shear dips.

Shear width showed greater correlation with the amount of energy absorbed. The fractured surface topography of the vertical specimens reflects the importance of greater ductility on notch toughness, i.e., ~51% greater shear width, ~35% greater shear lip height, ~18% shear length, and ~15% greater roughness in the flat fracture region than in the horizontal specimens.

The notch toughness of EB-PBF Ti-6Al-4V is well correlated with the hardness and tensile properties, i.e., tensile toughness and ductility.

## Figures and Tables

**Table 3:** Average roughness parameters of the fracture surfaces, excluding the shear area.

Orientation	Sa (μm)	Sq (μm)	Sz (μm)	Sp (μm)	Sv (μm)
HS	18.1	24.2	263.8	139.6	124.1
HT	19.1	26.1	375.8	200.3	175.5
Vertical	21.4	28.8	346.8	182.6	164.2

**Table 4:** Correlation of fractured surface features with energy absorption (J).

Features	Shear Width (mm)	Max Continuous Shear Lip Length (mm)	Max Shear Lip Height (mm)	Average Roughness, Sa (μm)	Root Mean Roughness, Sq (μm)
Correlation with Horizontal	−0.16	0.13	−0.30	0.33	0.41
Correlation with Vertical	0.59	0.41	−0.32	0.21	0.16
Correlation (Combined)	0.43	0.31	0.04	0.36	0.30

## Data Availability

The original contributions presented in this study are included in the article. Further inquiries can be directed to the corresponding authors.
